# Combination Analgesic Development for Enhanced Clinical Efficacy (CADENCE Trial): Study Protocol for a Double-Blind, Randomized, Placebo-Controlled Crossover Trial of an Alpha-Lipoic Acid – Pregabalin Combination for the Treatment of Fibromyalgia Pain

**DOI:** 10.2196/resprot.8001

**Published:** 2017-08-04

**Authors:** Ian Gilron, Dongsheng Tu, Ronald Holden, Tanveer Towheed, Elizabeth Vandenkerkhof, Roumen Milev

**Affiliations:** ^1^ Queen's University Department of Anesthesiology and Perioperative Medicine Queen's University Kingston, ON Canada; ^2^ Queen's University Kingston, ON Canada

**Keywords:** fibromyalgia, alpha-lipoic acid, antioxidant, pregabalin, anticonvulsant

## Abstract

**Background:**

Fibromyalgia is a clinical disorder commonly presenting with chronic widespread pain as well as sleep disturbance, fatigue, depression, and cognitive dysfunction. There is an urgent need for treatment strategies that provide better pain relief and fewer adverse effects (AEs). Efforts to develop rational combinations of specific fibromyalgia treatments have demonstrated potential for measurable improvements in pain relief, quality of life, and health care utilization. More than half of fibromyalgia patients receive 2 or more analgesics but current combination use is based on limited evidence. As an early proof-of-concept project from the Canadian Institutes of Health Research–Strategy on Patient-Oriented Research Chronic Pain Network, this trial protocol is expected to advance the field by rigorously evaluating a new treatment combination for fibromyalgia.

**Objective:**

We will test the hypothesis that analgesic combinations containing at least one nonsedating agent would be as safe but more effective than either monotherapy because of additive pain relief without increasing overall AEs. Pregabalin (PGB), a sedating anticonvulsant, is proven effective for fibromyalgia, and the antioxidant, alpha-lipoic acid (ALA), one of the only nonsedating systemic agents proven effective for neuropathic pain, is currently being evaluated in fibromyalgia. Thus, we will conduct a clinical trial to compare a PGB+ALA combination to each monotherapy for fibromyalgia.

**Methods:**

Using a double-blind, double-dummy, crossover design, 54 adults with fibromyalgia will be randomly allocated to 1 of 6 sequences of treatment with PGB, ALA, and PGB+ALA combination. During each of 3 different treatment periods, participants will take 2 sets of capsules containing (1) ALA (or placebo) and (2) PGB (or placebo) for 31 days, followed by an 11-day taper/washout period. The primary outcome will be mean daily pain intensity (0 to 10 scale) at maximal tolerated doses (MTDs) during each period. Secondary outcomes, assessed at MTD, will include global improvement, adverse events, mood, and quality of life.

**Results:**

This trial attained ethics approval March 6, 2017 (Queen’s University Health Sciences and Affiliated Teaching Hospitals Research Ethics Board protocol number ANAE-313-17), and recruitment is set to start in August 2017.

**Conclusions:**

This trial will provide rigorous evidence comparing the efficacy of a PGB-ALA combination to PGB alone and ALA alone in the treatment of fibromyalgia.

**Trial Registration:**

International Standard Randomized Controlled Trial Number ISRCTN14939460; https://www.isrctn.com/ ISRCTN1493946 (Archived by WebCite at http://www.webcitation.org/6sFqAjxkt)

## Introduction

Chronic pain affects 20% to 25% of the population [1] and is one of the most common reasons to see a health care provider and to miss work [2]. In North America alone, chronic pain costs over $650 billion in health care and lost productivity [3]. Fibromyalgia is a complex clinical disorder characterized by chronic widespread pain that is also associated with sleep disturbance, fatigue, irritable bowel syndrome, depressed mood, and, possibly cognitive dysfunction [4,5]. Patients suffering with fibromyalgia very frequently report functional disability and impaired quality of life [6]; furthermore, fibromyalgia is a common disorder estimated to affect 1.6% of men and 4.9% of women [7].

Hundreds of randomized controlled trials (RCTs) have evaluated various drug (eg, nonsteroidal anti-inflammatory drugs, antidepressants, opioids, and anticonvulsants) and nondrug (eg, exercise, acupuncture, cognitive behavioral therapy) therapies for fibromyalgia [6]. In addition to exercise and cognitive behavioral therapy, pharmacotherapy remains an important treatment for fibromyalgia. Evidence-based treatment recommendations from various groups including the European League Against Rheumatism and Canadian Pain Society have included amitriptyline, cyclobenzaprine, tramadol, gabapentin/pregabalin, fluoxetine, and duloxetine [8-10].

Available drugs used for fibromyalgia reduce pain on average by only 25% to 40%, and meaningful relief occurs in only 40% to 60% of patients, in part due to incomplete efficacy as well as commonly encountered dose-limiting adverse effects (AEs) (eg, sedation, cognitive dysfunction, and dizziness). Combining two drugs with different pharmacological mechanisms has the potential to provide superior relief over monotherapy without increasing side effects [11]. A recent trial has demonstrated greater analgesic efficacy with a pregabalin-duloxetine combination versus either monotherapy without an increase in side effect profile [12]. Although this was a positive finding, the additive benefit was submaximal because these two agents cause some similar AEs, and doses must be reduced during combination therapy to maintain safety and tolerability.

Thus, we hypothesize that analgesic combinations containing at least one nonsedating agent would provide even greater additive benefits because of additive pain relief but nonadditive AEs. Both pregabalin (PGB) and alpha-lipoic acid (ALA) are approved by Health Canada and proven for the treatment of neuropathic pain [13,14]. Based on a rationale for the use of ALA to treat fibromyalgia pain [15], a placebo-controlled RCT of ALA monotherapy is currently under way in this population [ISRCTN58259979].

An important pharmacological mechanism of PGB is the blockade of α-2-δ subunits of N-type voltage gated calcium channels, resulting in decreased calcium influx and neurotransmitter release [16,17]. ALA has been studied in both preclinical and clinical neuropathic pain conditions. In a rat model of streptozocin-induced diabetes, ALA delayed the onset of polyneuropathy [18]. Mechanistic studies suggest decreased nociceptive sensitivity by inhibition of T-type calcium (Cav3.2) channels [19], distinct from that of PGB which inhibits N-type calcium channels [17], suggesting potential for synergy at these different sites of action.

At least 16 trials of over 1320 patients have reported reductions in pain and other symptoms [13,20] and a recent meta-analysis reported a number needed to treat of 6.3 [13]. Also, 1 trial reported improvement in neuropathic pain symptoms after 4 years of treatment [21]. AEs of nausea, vomiting, headache, and vertigo have been reported in studies involving >1200 mg per day of ALA. There have also been rare reports of hypoglycemia (low blood sugar) in diabetic patients taking ALA and reporting symptoms of sweating, paleness, chills, headache, dizziness, and/or confusion. We identified only 1 study of a combination similar to ALA+PGB—ALA plus gabapentin (related to PGB) in the treatment of burning mouth syndrome [22]. Despite the study having major methodological flaws, greater benefit with this combination was suggested versus monotherapy, and AEs were reported overall as very mild [22].

Thus, our goal is to conduct a novel double-blind RCT to compare the combination of the anticonvulsant PGB with the nonsedating antioxidant ALA to each monotherapy for the treatment of pain in fibromyalgia.

## Methods

### Ethics

This study underwent ethics review and received a compliance notice by the Queen’s University Health Sciences and Affiliated Teaching Hospitals Research Ethics Board on March 6, 2017. This trial will be conducted at one site, Providence Care Hospital, Kingston, Ontario, Canada. This trial is registered with the International Standard Randomized Controlled Trial Number Registry [ISRCTN14939460].

**Figure 1:**
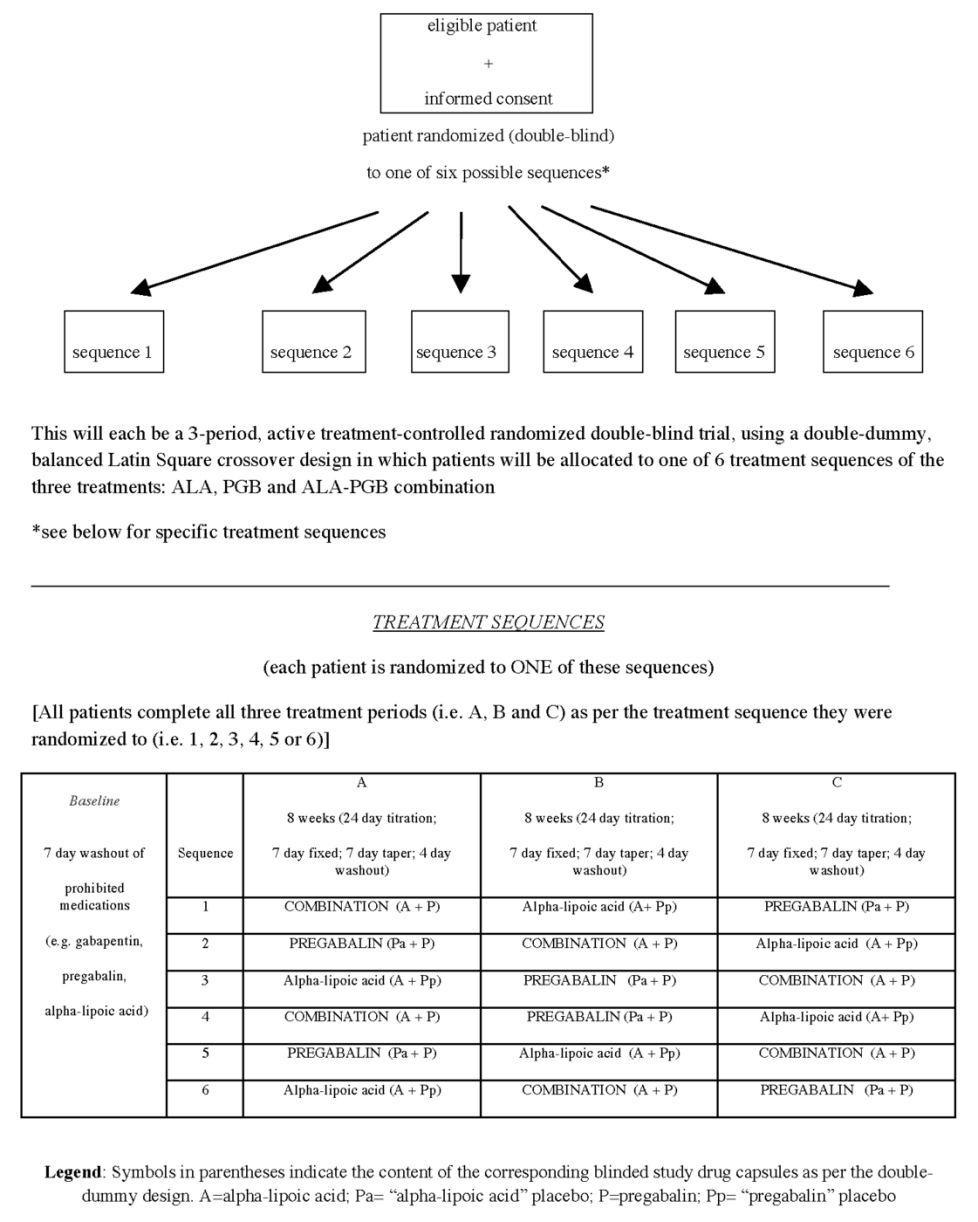
Trial design.

### Aims and Hypothesis

The objective of this trial is to compare the safety and efficacy of a PGB+ALA combination to each monotherapy in treating participants with fibromyalgia. Our primary hypothesis is that PGB+ALA has greater analgesic efficacy versus either monotherapy.

### Trial Design

**Figure 2:**
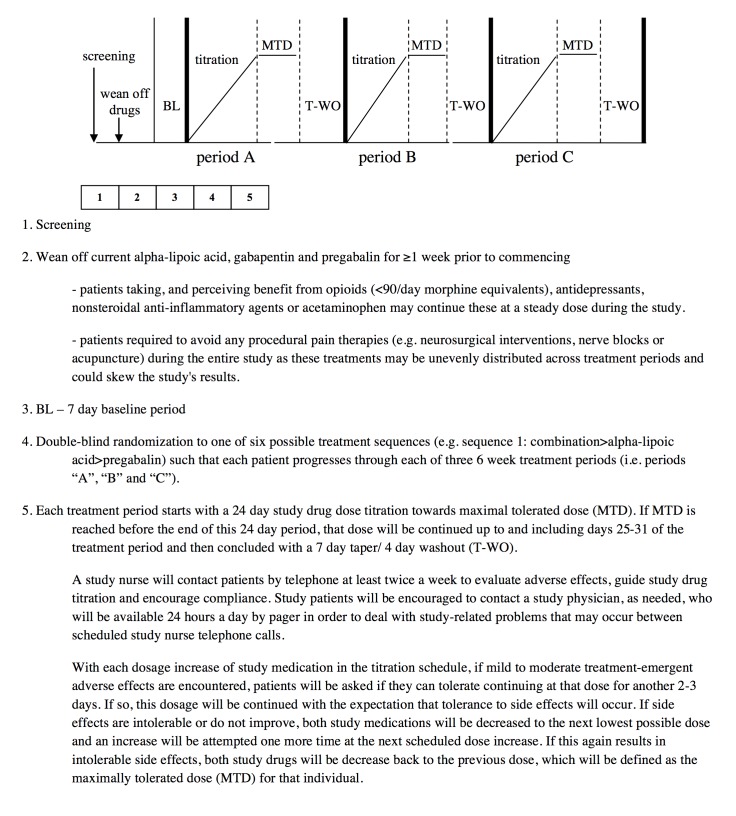
Trial design, continued.

We have designed a single-center, double-blind, double-dummy, randomized, controlled 3-period crossover trial comparing a PGB+ALA combination to monotherapy in treating fibromyalgia (Figures 1 and 2). This trial is compliant with Health Canada/International Conference on Harmonization guidelines and incorporates outcome measures recommended by the Initiative on Methods, Measurement, and Pain Assessment in Clinical Trials. Using a flexible dose titration, Latin Square crossover design, treatments will be titrated during each of 3 treatment periods to maximal tolerated dose (MTD) with primary and secondary trial analyses comparing the 3 treatments using end-of-period outcomes. Internal validity of our crossover design is supported by stability of fibromyalgia over time [23-26] and the risk of carryover from 1 period to the next is very low because each period is followed by an 11-day dose taper and drug washout, and the final MTD week for each period (from which the primary outcome is obtained) is separated from the next period’s final week by 7 weeks (ie, ≥20 half-lives of the drugs studied). Nevertheless, exploratory analyses will be conducted to identify if any low-order carryover effect does exist.

### Participant Allocation

As per the 3-period Latin Square crossover, patients are randomly allocated to 1 of 6 sequences of ALA, PGB, and combination (Figures 1 and 2). Before the trial, an independent pharmacist and biostatistician will prepare a concealed allocation schedule using a computer-generated block randomization process to randomly assign treatment sequences to a consecutive series of numbers within a block. Each patient will be assigned to the next consecutive number, and the corresponding sequence of medications will be dispensed. All study personnel will be blinded to the block sizes to preserve allocation concealment.

### Protecting Against Bias

Medications will be encapsulated (ALA: blue, PGB: gray) in an identical fashion across all periods. As per a double-blind, double-dummy design, patients will take both sets of medications so treatment conditions will be identical across all 3 treatment periods. Treatment codes will be generated by the investigational pharmacist and concealed until trial completion. In case of emergency, individual codes will be disclosed by an investigational pharmacist to a nonstudy clinician. A questionnaire completed by every participant at the end of each period will ask patients to guess the treatment they received to assess blinding.

### Participants

Men and women aged 18 years and older meeting the 2016 updated American College of Rheumatology diagnostic criteria for fibromyalgia [27] will be considered for recruitment following informed consent. See Textbox 1 for selection criteria.

**Selection criteria.**

Inclusion criteria:

- Adults aged 18 years and older
- Diagnosed with fibromyalgia using the 2016 updated American College of Rheumatology diagnostic criteria [27]
- Experience daily moderate pain (≥4/10 on a numerical rating scale) for at least 3 months
- Women of childbearing potential must have a negative serum beta–human chorionic gonadotropin test and are required to use a highly effective form of contraception while on trial
- Have the necessary abilities, visual acuity, and English language skills to complete questionnaires and pain diaries and to participate in telephone communication with study nurses to permit titration of the study drugs

Exclusion criteria:

- Presence of a painful condition, including inflammatory rheumatic disease, more than 50% as severe as but distinct from fibromyalgia
- Women who are pregnant or lactating
- Women of childbearing potential not using adequate contraceptives
- End-stage kidney or liver disease
- Unstable cardiovascular disease (myocardial infarction within the preceding year, unstable angina, or congestive heart failure) or clinically relevant abnormal 12-lead electrocardiogram
- Any poorly controlled medical condition that, in the opinion of the investigator, would interfere with proper conduct of the trial
- Severe depression, as determined by a Beck Depression Inventory–II score of 29 or more; suicidal ideation, as determined by a Beck Depression Inventory–II item 9 score of 2 or more; or any current major psychiatric disorder (eg, schizophrenia, bipolar disorder) that is not well controlled
- Hypersensitivity to any of the study medications
- Any current alcohol or drug abuse or dependence (except nicotine and caffeine). Participants with a history of abuse or dependence with more than 1 year of abstinence may be considered for inclusion
- Those taking more than 90 mg morphine equivalents per day

### Study Interventions

**Figure 3:**
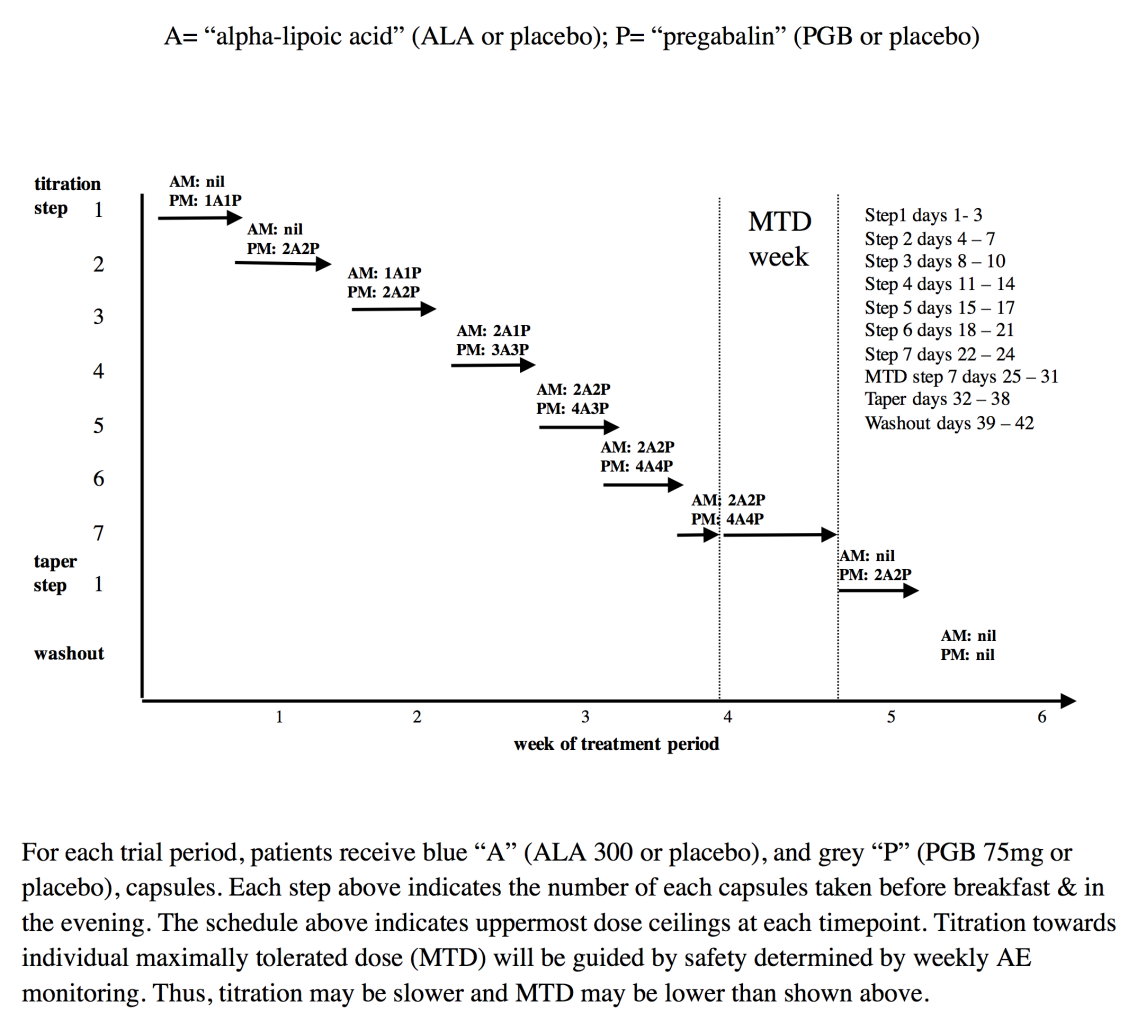
Study drug schedules.

During each of 3 trial periods, using a double-blind randomized crossover design, patients will receive 2 sets of capsules (Figure 3): (1) blue capsules (ALA 300 mg or placebo) and (2) gray capsules (PGB 75 mg or placebo). During the combination period, blue will contain ALA and gray will contain PGB. During the ALA alone period, blue will contain ALA and gray will contain placebo. During the PGB alone period, blue will contain placebo and gray will contain PGB.

Consenting patients on ALA or PGB (or gabapentin) pretrial will agree to be weaned gradually for a washout of at least 7 days. Co-interventions: patients taking and perceiving benefit from opioids (<90 mg morphine equivalents), antidepressants (tricyclic, selective serotonin reuptake inhibitors or serotonin-norepinephrine reuptake inhibitors), nonsteroidal anti-inflammatory drugs, or acetaminophen may continue these at a steady dose for the entire study. Any cognitive behavioral therapy or exercise programs may continue only if they can be scheduled evenly across all treatment periods. Research staff will monitor and advise patients weekly about prohibited co-interventions throughout the study. A thorough understanding of the threats to validity of using forbidden co-interventions (gabapentin, PGB, ALA, and any other newly initiated analgesic intervention) is heavily emphasized to participants. Patients will not be allowed to start new cognitive behavioral therapy or exercise programs after study initiation and must avoid any procedural therapies (eg, nerve blocks or acupuncture) during the entire study. Any pain exacerbations that in the opinion of the patient warrant initiation of a new therapy would necessitate trial discontinuation and immediate weaning from study medications, but these patients would still be included in the trial analyses.

### Dose Titration

Study medication will follow a flexible dose titration to MTD to balance tolerability and relief, with regularly weekly calls by research personnel. This means that doses of study medication will not be further increased if intolerable AEs are encountered at higher doses or if “a lot” or “complete” pain relief is achieved. The MTD fixed dose week will be from days 25 to 31. However, if MTD is reached before day 25, that MTD dose will continue up to and including the day 25 to 31 period. The MTD fixed dose week will be followed by a 7-day dose taper and 4-day complete washout. Daily pain ratings will be completed throughout the trial. During dose taper and washout periods only, patients may take acetaminophen ≤8 tablets per day (325 mg per tablet) as needed. This rescue medication is very unlikely to affect the primary outcome measure of pain intensity during the MTD phase of each treatment period.

### Trial Duration and Follow-Up Frequency

**Figure 4:**
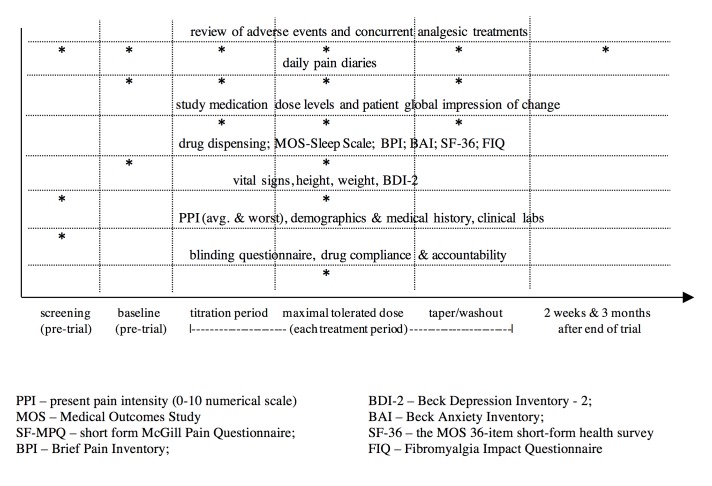
Schedule of assessments.

Each of the 3 treatment periods will be 6 weeks, for a total trial duration of 18 weeks. The nurse will phone patients weekly to evaluate AEs, guide drug titration, and encourage compliance. Patients will be seen in clinic at the end of each treatment period for assessment of vital signs and measurement of secondary outcomes (Figure 4). Patients will be followed up by phone 2 weeks and 3 months after trial completion (including patients who were withdrawn from the trial prematurely) to document any subsequent AEs.

### Outcome Measures and Safety Assessment

The primary outcome is mean daily pain (0 to 10 numerical rating scale with 0=no pain and 10=worst pain imaginable), rated 3 times daily (8 AM, 4 PM, and 8 PM) and averaged over the MTD fixed dose week (days 39 to 45) of each period. Secondary outcomes include daily pain at other timepoints, the MTDs of PGB and ALA, frequency and severity of AEs and patient global impression of change [28], short form McGill Pain Questionnaire [29], Fibromyalgia Impact Questionnaire [30], Brief Pain Inventory [31], Beck Depression Inventory-II [32], Beck Anxiety Inventory [33], Short Form-36 health survey [34], blinding questionnaires, and acetaminophen consumption. Timing of outcome assessments is described in Figure 4. Patient safety will be ensured by vigilant AE assessment and judicious drug titration. Any occurrences of major AEs will be tracked as secondary outcomes and also reported to the Queen’s Ethics Board, Health Canada. Assessment and reporting of AEs will adhere to Consolidated Standards for Reporting Trials recommendations [35].

### Sample Size

Based on previous estimates of within-patient variation, *s*=2.45, from a previous study in fibromyalgia [36], we calculate that a sample of 55 trial completers would provide an 80% chance of detecting (alpha of .05) a mean treatment difference of 1 point (0 to 10 scale). For a sample size divisible by 6, the number of treatment sequences, we adjusted the sample size to 54 patients.

### Statistical Analyses

Analyses for this trial are based on the null hypothesis of no difference between PGB, ALA, and PGB+ALA, and the alternative hypothesis that at least 2 treatments are different. When a patient contributes data from only 1 period, sensitivity analyses including all patients will also be performed by assuming some reasonable but extreme values for the remaining periods. All patients receiving at least 1 dose of a drug will be included in the safety analyses.

#### Primary Outcome Analysis

The primary outcome—mean daily pain from the last 7 days (at MTD) of each treatment period—will be calculated as an average of pain scores as recorded in the pain diary if more than 50% of the information (ie, at least 4 days) is not missing. Otherwise, mean daily pain will be treated as missing. This is based on the half rule often used to summarize repeated responses, which has proven unlikely to introduce bias to trial results [37]. Sensitivity analyses based on the average of all available pain scores will also be performed to confirm the results of the primary analysis. Although carryover effects are unlikely, we recognize this possibility. Therefore, a linear mixed model with sequence, period, treatment, and the first order carryover term as fixed effects and patient as a random effect [38] will be used to test for differences among the 3 treatments and to estimate the least square mean of the mean daily pain intensity for each treatment, adjusting for carryover as well as period effects (ie, stability of pain levels). The following 3 pair-wise comparisons will be performed based on the least square means and standard deviations from the linear mixed model: combination versus ALA alone, combination versus PGB alone, and ALA alone versus PGB alone. Sensitivity analyses will be performed using a pattern-mixture model [39] based on patterns of missing data so as to check the robustness of results in the case that data may not be missing at random. A Fisher's least significant difference [40] procedure will be used to adjust the *P* values for these 3 comparisons.

#### Secondary Analyses

Secondary outcomes will be analyzed similarly except that only one measurement is analyzed in the last week for the singular measures (ie, final week questionnaires) and the scoring algorithms developed for the Brief Pain Inventory, Beck Depression Inventory-II, and Short Form-36 will be first used to derive the subscales or domains within these instruments, and the scores on these subscales or domains will be used as response variables in the linear mixed model analysis.

As with many other analgesic trials that allow concomitant medications, treatment group comparisons are made with the assumption in the setting of randomization of an equal distribution of concomitant medications across participants. Nevertheless, our trial analysis will further conduct exploratory analyses, as we have done on our previous trials, to investigate the possibility that concomitant analgesic medications have an important effect on the trial results.

## Results

Participant recruitment is expected to begin in August 2017. This trial was awarded external peer-reviewed funding by the Canadian Institutes of Health Research–Strategy on Patient-Oriented Research–Canadian Pain Network in August 2016.

## Discussion

Fibromyalgia continues to be difficult to manage, and current treatments provide only partial relief often at the risk of disabling AEs. To the best of our knowledge, this proposed trial is the first to compare the combination of an anticonvulsant with an antioxidant to treat fibromyalgia. Because ALA and PGB have different AE profiles, we expect their combination to provide superior analgesic efficacy in fibromyalgia without increasing AEs.

Possible threats to trial completion include challenges with participant recruitment, noncompliance, protocol violations, and early dropouts. However, we are confident that the proposed trial design and our experience with recent and previous RCTs will minimize these concerns. Noncompliance, protocol violations, and early dropouts will be minimized by the crossover design as well as thorough patient teaching and careful follow-up of trial participants.

Given the urgent need for improved fibromyalgia treatments that provide better pain relief with better safety and tolerability, this trial will provide rigorous evidence for a potentially improved treatment strategy for fibromyalgia.

## Abbreviations

AE: adverse effect
ALA: alpha-lipoic acid
MTD: maximal tolerated dose
PGB: pregabalin
RCT: randomized controlled trial

## Appendix

Multimedia Appendix 1 Peer reviewer comments (CIHR-reviews-CADENCE-funded).
