# Supplementary material for: Combination Analgesic Development for Enhanced Clinical Efficacy (CADENCE Trial): Study Protocol for a Double-Blind, Randomized, Placebo-Controlled Crossover Trial of an Alpha-Lipoic Acid – Pregabalin Combination for the Treatment of Fibromyalgia Pain
Source: JMIR Res Protoc. 2017 Aug 4;6(8):e154. doi: 10.2196/resprot.8001 (PMC5705061; doi:10.2196/resprot.8001)
Supplement: Multimedia Appendix 1 [file resprot_v6i8e154_app1.pdf]

Review Type/Type d'évaluation: Committee Member 1/Membre de comité 1
Name of Applicant/Nom du chercheur: GILRON, Ian
Application No./Numéro de demande: 341067
Agency/Agence: CIHR/IRSC
Competition/Concours: 2015-03-02 Operating Grant/Subvention de fonctionnement
Committee/Comité: Randomized Controlled Trials/Essais contrôlés randomisés
Title/Titre: CADENCE: Combination Analgesic Development for Enhanced Clinical Efficacy

---

Assessment/Évaluation:

This is the third submission of a previous proposal.

1. **Research Approach:**

The overall objectiveofthisapplication istocompare the efficacy andadverseeffects associated with thecombinationofthesedating anticonvulsantdrug,pregabalin(PGB),and the non-sedatingantioxidantagent,alpha-lipoicacid (ALA) versus each agent alone for the treatment of chronic neuropathic pain.

The primary outcome will be daily pain from the maximal tolerable dose (day 39-45) for each treatment period using a numerical rating scale. The applicants propose a 3-period,active treatment-controlled randomized double-blindtrial,using a double-dummy,balanced Latin Square crossover design in which patients will be allocated to one of 6 treatment sequences of the three treatments: ALA, PGBand ALA-PGBcombination. Each of the three treatment periods will be 8 weeks induration for a total of 24 weeks per patient. The applicants propose a sample size of 84 patients to allow for the cross-over design and loss to follow up. They project that the trial will take 34 months to complete.

1. The rationale for this study is that chronic pain is very common and has significant social and economic impact and 50% of patients with chronic neuropathic pain use combination therapies that are not well studied or have significant side effects. The investigators postulate that using combination therapy of a non-sedating agent along with a sedating agent will maximize efficacy while minimizing unwanted side effects. ALA is the only non-sedating drug shown to be effective in neuropathic pain. PGB has been shown to be very effective in neuropathic pain but is quite sedating at the higher more effective doses. The applicants provide a compelling argument as to why this specific combination needs to be studied.
2. Concerns had been previously raised as to why a placebo group was not used but this reviewer agrees with the applicants that a placebo group would not be ethical for a combination therapy pain trial.
3. The use of a cross-over trial versus a parallel design trial is justified by the postulate that the specific mechanism for pain in individual patients differs and that therefore it would make more physiological sense for each patient to serve as their own control. In addition, this design would obviously decrease the needed sample size.
4. There has been significant discussion as to whether a minimal clinically important difference of 1 is justified. Based on the rationale provided by the applicants and the pain literature, this reviewer feels that it is.
5. It was previously raised that there had not been enough background work to justify this particular

Review Type/Type d'évaluation: Committee Member 1/Membre de comité 1
Name of Applicant/Nom du chercheur: GILRON, Ian
Application No./Numéro de demande: 341067
Agency/Agence: CIHR/IRSC
Competition/Concours: 2015-03-02 Operating Grant/Subvention de fonctionnement
Committee/Comité: Randomized Controlled Trials/Essais contrôlés randomisés
Title/Titre: CADENCE: Combination Analgesic Development for Enhanced Clinical Efficacy

---

Assessment/Évaluation:

study, however, I believe that between the available literature and the collateral work in this general area, this study is justified.

6. A minor concern is the choice to use an 8 week treatment period instead of a 12 week one. While the applicants explain that 8 weeks is a compromise between the time needed to show a difference versus extending the length of the trial for participants, I still feel that given the literature supporting a 12 week trial period per regimen that the applicants should seriously consider changing this.

Originality of proposal

This particular combination of therapies (and mechanisms of action) has not been studied before and has the potential to be very effective.

Applicant

The PI is a professor in the Departments of Anesthesia and Biomedical and Molecular Sciences at the University of Toronto and is the current recipient of the SEAMO Clinician Scientist Development Program Research Salary Award and a Mid-Career Investigator Award Research award from CIHR. He currently holds 9 grants and is the PI on 6 all of which are related to pain research. He has held an additional 5 grants in the past 5 years. He has supervised a large number of post docs and students and has received both funding and awards for his mentorship. He has given a large number of invited talks throughout the world on pain management and is clearly considered an expert in the field.

The co-applicants on this proposal have expertise in psychology, psychiatry, epidemiology and biostatistics and neurosciences. This team has worked together on other projects in the past and clearly has the expertise to conduct this study.

Environment for research

The applicant clearly has an excellent environment for research as demonstrated by his ability to perform and

Review Type/Type d'évaluation: Committee Member 1/Membre de comité 1
Name of Applicant/Nom du chercheur: GILRON, Ian
Application No./Numéro de demande: 341067
Agency/Agence: CIHR/IRSC
Competition/Concours: 2015-03-02 Operating Grant/Subvention de fonctionnement
Committee/Comité: Randomized Controlled Trials/Essais contrôlés randomisés
Title/Titre: CADENCE: Combination Analgesic Development for Enhanced Clinical Efficacy

Assessment/Évaluation:

complete RCTs.

-

Impact of the research

Chronic pain is a huge problem. However, this study focuses on one specific subgroup of this population and one specific combination of therapy. The intervention has the potential to significantly impact the lives of those with chronic neuropathic pain but the broader societal impact is not clear.

Budget

Although the cost per patient is rather high, the budget is well justified and the overall cost of the project is very reasonable for the amount of work required.

Review Type/Type d'évaluation: Committee Member 2/Membre de comité 2
Name of Applicant/Nom du chercheur: GILRON, Ian
Application No./Numéro de demande: 341067
Agency/Agence: CIHR/IRSC
Competition/Concours: 2015-03-02 Operating Grant/Subvention de fonctionnement
Committee/Comité: Randomized Controlled Trials/Essais contrôlés randomisés
Title/Titre: CADENCE: Combination Analgesic Development for Enhanced Clinical Efficacy

---

Assessment/Évaluation:

CADENCE: Combination analgesic development for enhanced clinical efficacy
Principal Applicant: Ian Gilron
Synopsis:
Purpose
The purpose is to compare pregablin/alpha-lipoic acid combination to both pregablin (PGB) and to alphalipoic acid (ALA) for the treatment of diabetic neuropathic pain. The researchers also want to compare PGB to ALA. This will be accomplished by randomized, double blind, three period crossover study.
Hypothesis
Combination of PGB-ALA has superior efficacy to either PGB or ALA individually
Objectives
Primary: compare PGB/ALA to either PGB or ALA individually on numerical rating scale of 0-10
Secondary: compare PGB to ALA
Outcomes include pain scores at other time points not in the maximal tolerated dose week, adverse effects, global impression of change, short form McGill Pain Quest., Beck Depression Inventory, Neuropathic Pain Symptom Inventory, Brief Pain Inventory, Beck Anxiety Inventory, SF-36, acetaminophen consumption, blinding questionnaire, Medical outcomes study sleep scale.

Progress
The researchers have extensive experience in running trials with similar methodology. (nortriptyline/morphine, gabapentin/morphine, nortriptyline/gabapentin)
They have written review articles on use of medications in neuropathic pain.
Assessment
Research approach
The research question is clear, as are secondary objectives. The literature is well reviewed and relevant. This includes previous combination studies, studies on the bot PGB and ALA. Outcome measures to be used. The design is appropriate. The justification of a crossover study rather than parallel approach is well explained. The follow up phone calls (two times a week) to maintain compliance questions the generalizability of the results. The researchers justify this by quoting studies that have confirmed their results in the past. However one quoted paper (114) did not find significant results and the study itself was an RCT after failure of monotherapy. Another quoted study (34) had a much higher drop out rate in combination therapy which was not addressed in the analysis. Thus generalizability of study results may be less clear. However, there is a need for accurate information and compliance.
The feasibility of the study and outline is well drawn and achievable.
The difficulties which are mainly compliance is considered by increasing research coordinator calls (2

Review Type/Type d'évaluation: Committee Member 2/Membre de comité 2
Name of Applicant/Nom du chercheur: GILRON, Ian
Application No./Numéro de demande: 341067
Agency/Agence: CIHR/IRSC
Competition/Concours: 2015-03-02 Operating Grant/Subvention de fonctionnement
Committee/Comité: Randomized Controlled Trials/Essais contrôlés randomisés
Title/Titre: CADENCE: Combination Analgesic Development for Enhanced Clinical Efficacy

---

Assessment/Évaluation:

times/week). This is at the cost of generalizability.
The balance is between generalizability and feasibility. The researchers have leaned towards feasibility, knowing this is a difficult area of study.
Originality
The research is original in combining two medications with dissimilar side effect profiles. As pain management is usually multimodal, this is important information to guide practice.
Applicants:
The research team is strong. The principal applicant has successfully completed similar trials. The team members each bring particular strengths.
Budget:
Appears appropriate
Strengths:
The team is very strong with a solid record. It has the ability to complete the trial as proposed. The area of research is important and the possible impact on management is substantial. It is difficult to produce high quality trials in chronic pain management.
Inclusion criteria are clear. Treatment allocation and study reporting is clear.
Excellent measurement of recruitment period base on population and previous experience. Thus trial is possible within timeframe outlined.
Weakness:
The trial period of 8 weeks is not sufficient. Although important for maintaining compliance (as the applicants appropriately describe), it remains insufficient. There is no justification that the results of a standard 12 trial is equivalent to 8 weeks.
The applicants are looking for statistical significance, however clinical significance will change practice patterns. A change of 0.5-1 on a VAS is not sufficient to affect clinical change.
How will the maximal tolerated dose be evaluated? How will the study coordinator determine that this threshold has been reached and that the side effects are medication related?
There are many outcome measures reported. There is no information on how they will impact the reporting of the results or outcomes.
The sample size is not based on clinically important changes in pain but on statistically significant measures.
The sample size is also based on “there is at least one pair of study treatments that are different”. This outcome is different than the primary research question.
It is unclear how the researchers will account for the participants who are noncompliant o do not fill out their diaries (to more than 50%), How will a sensitivity analysis be interpreted.

Review Type/Type d'évaluation: Committee Member 3/Membre de comité 3
Name of Applicant/Nom du chercheur: GILRON, Ian
Application No./Numéro de demande: 341067
Agency/Agence: CIHR/IRSC
Competition/Concours: 2015-03-02 Operating Grant/Subvention de fonctionnement
Committee/Comité: Randomized Controlled Trials/Essais contrôlés randomisés
Title/Titre: CADENCE: Combination Analgesic Development for Enhanced Clinical Efficacy

Assessment/Évaluation:

This is a three period cross-over trial lasting 24 weeks (with twice-weekly contact with patients) looking at ALA, pregabalin and their combination. Interest is in all three pairwise comparisons. While cross-over trials are difficult to conduct and patient dropout can compromise validity, the study team has successfully completed cross-over trials before, which have been published in high-impact journals (e.g. NEJM, Lancet). The investigators position it as a proof-of-concept trial (in places referred to as a phase IIb trial), but its influence on clinical practice is also discussed. It is not entirely clear whether the investigators believe that this trial has the potential to produce evidence that warrants a change in practice without further research, or whether it is a precursor to a more definitive trial.

Major concerns
My main concern is dropout. The investigators estimating 30% dropout based on previous experience (81 patients to end up with 54 patients). This is likely realistic, but is also high and raises questions about generalizability of the results.
There is some inconsistency in describing how missing data will be handled. The analysis plan states that "All patients will be included in the analyses. When data from only one period are available, sensitivity analysis including all patients will also be performed…" Which statement is correct? Since analysis will use a linear mixed model, there should be no problem including all patients even without assuming extreme values for other periods.
Analysis will be based on mean pain score from the last 7 days, however as different patients will have different numbers of measurements, this will result in an outcome that is heteroscedastic (means based on more measurements will have smaller variances), and hence run counter to the assumptions of the linear mixed model. Is there any reason why one cannot just use the individual pain scores for each day, for each patient, without averaging?

Minor points
Section 2.16 states that the aim is to test the null hypothesis that there is no difference in pain intensity for any of the treatments vs. the alternative that there is at least one pair of treatments that are different. However, the analysis plan describes pairwise testing, and this is also what the sample size calculation is based on. Pairwise testing seems appropriate, but consistency in the protocol as to the main objective would be helpful.
The summary cites NNTs but does not say what the outcome is, i.e. number needed to treat to achieve *what*? This is not described until the main proposal.
The fact that pain trials reveal that statistically significant differences vary between 0.5 to 1 points is not adequate evidence that 1 point is a clinically meaningful difference (response to reviews, proposal page 10).

**Review Type/Type d'évaluation:**	Committee Member 3/Membre de comité 3
**Name of Applicant/Nom du chercheur:**	GILRON, Ian
**Application No./Numéro de demande:**	341067
**Agency/Agence:**	CIHR/IRSC
**Competition/Concours:**	2015-03-02 Operating Grant/Subvention de fonctionnement
**Committee/Comité:**	Randomized Controlled Trials/Essais contrôlés randomisés
**Title/Titre:**	CADENCE: Combination Analgesic Development for Enhanced Clinical Efficacy

Assessment/Évaluation:

However I agree that if the mean difference is 1 point then a significant proportion of patients will experience a two-point improvement (based on the fact that the sd of the difference between time periods is 2.3 (Appendix 13 and Gilron 2005; in fact, I estimate that 33% of patients would experience an improvement of 2 points or more while 10% would experience a deterioration of 2 points or more).
